# Financial toxicity due to breast cancer treatment in low- and middle-income countries: evidence from Vietnam

**DOI:** 10.1007/s00520-021-06210-z

**Published:** 2021-04-16

**Authors:** Tran Thu Ngan, Hoang Van Minh, Michael Donnelly, Ciaran O’Neill

**Affiliations:** 1 Centre for Public Health, Queen’s University Belfast, Belfast, UK; 2 Centre for Population Health Sciences, Hanoi University of Public Health, Hanoi, Vietnam

**Keywords:** Breast cancer, Financial toxicity/hardship, Cost of treatment, Vietnam, LMICs

## Abstract

**Background:**

This study examined the financial toxicity faced by breast cancer (BC) patients in Vietnam and the factors associated with the risk and degree of that toxicity.

**Methods:**

A total of 309 BC patients/survivors completed an online survey (*n*=209) or a face-to-face interview (*n*=100) at two tertiary hospitals. Descriptive statistics and *χ*^2^ tests were used to identify and analyse the forms and degree of financial toxicity faced by BC patients/survivors. A Cragg hurdle model assessed variation in risk and the degree of financial toxicity due to treatment.

**Results:**

41% of respondents faced financial toxicity due to BC treatment costs. The mean amount of money that exceeded BC patients/survivors’ ability to pay was 153 million Vietnamese Dong (VND) ($6602) and ranged from 2.42 million VND to 1358 million VND ($104–58,413). A diagnosis at stage II or III of BC was associated with 16.0 and 18.0 million VND (~$690–777) more in the degree of financial toxicity compared with patients who were diagnosed at stage 0/I, respectively. Being retired or married or having full (100%) health insurance was associated with a decrease in the degree of financial toxicity.

**Conclusions:**

A significant proportion of Vietnamese BC patients/survivors face serious financial toxicity due to BC treatment costs. There is a need to consider the introduction of measures that would attenuate this hardship and promote uptake of screening for the reduction in financial toxicity as well as the health gains it may achieve through earlier detection of cancer.

**Supplementary Information:**

The online version contains supplementary material available at 10.1007/s00520-021-06210-z.

## Background

Breast cancer (BC) among Vietnamese women has the highest age-standardized incidence rate (26.4/100,000 in 2018) of all cancers [1]. BC is also the leading cause of cancer-related death in women, accounting for 13.9% of deaths [1]. The impact of BC extends beyond morbidity and mortality, however, giving rise to significant financial burden and distress. As noted by one BC patient, ‘People with money think about dying because of the disease. People without money think about dying because of not having money’ [2]. The patient-level impact of the cost of cancer care has been referred to as ‘financial toxicity’ which is consequence of both objective financial burden and subjective financial distress [3, 4]. Studies of this issue have increased in recent years though most evidence comes from high-income countries, especially from the USA [3]. Research from low- and middle-income countries (LMICs) is limited although patients in these countries are more financially vulnerable.

(Breast) Cancer can be an expensive disease to treat. The 1-year incidence of ‘financial catastrophe’ due to cancer treatment in Vietnam was 68% which was the highest among 10 countries in ASEAN—the Association of Southeast Asian Nations [5]. The greater financial burden in Vietnam may be due to three main reasons. Firstly, 49.5% of women with BC are diagnosed at late stages (stage III or IV) [6] which is associated with 95 to 109% higher treatment costs compared to stage I [7]. Secondly, in 2016, 81.7% of Vietnamese had health insurance (HI) meaning nearly 20% of the 95 million population will have to face 100% out-of-pocket (OOP) expenses should they be treated for BC [8]. Even when in possession of HI, women often still have to pay coinsurance equal to 20% of the health care cost (insurance covers the remaining 80%) which can still represent a significant financial outlay. Moreover, HI does not typically cover several expensive targeted drugs and procedures such as trastuzumab (HI covers 60%), pertuzumab and breast reconstruction surgery (are not covered by HI) where the cost must be met wholly OOP [6, 9, 10]. Thirdly, oncology hospitals providing tertiary treatment for BC are only located in a few large cities in Vietnam. This can give rise to significant direct non-medical costs (e.g. transportation and living expenses) for the patients and their families that must be borne OOP [6, 9].

In a longitudinal study of 309 BC patients with 12 months follow-up, 71.8% of households with BC patients faced catastrophic health expenditure (household’s total OOP is equal or exceed 40% of household’s income) [11]. Moreover, approximately half of non-poor households (49.2%) were impoverished by the OOP expenses for BC treatment (i.e. the households fell below the country’s poverty line after paying for all direct medical costs) [11]. While making a useful contribution to the literature this study did not examine how patients funded the OOP, how far into financial hardship the families of patients were driven by care costs, and what factors explained variation in financial toxicity’s risk or degree. Only one small qualitative study (13 patients) has examined the consequences of high OOP for BC care in Vietnam. This found that some patients had to sell their houses, take a loan, delay the treatment or even stop taking the treatment [2].

There is an urgent need to improve understanding about the impact of BC in Vietnam including its financial impact and the factors that are associated with differential experiences in this. The aim of this study was to assess the risk and degree of financial toxicity experienced by patients due to BC care costs as well as the factors associated with that risk and severity level.

## Methods

### Study design and participants

Data reported in this paper came from a larger study which examined OOP expenses on BC diagnosis and ‘initial treatment’ (defined as care which starts right after the diagnosis and normally includes surgery, radiotherapy and systemic therapies and often lasts up to 9 months [12, 13]). Data collection was undertaken in 2019 through an online survey and a hospital-based survey at two tertiary hospitals (Hanoi Oncology Hospital and Oncology Center of Hue Central Hospital). Respondents included both BC patients (who were undergoing initial treatment) and BC survivors (who had finished initial treatment). Financial well-being up to/at the point of interview (i.e. their current stage in the treatment pathway) was recorded for BC patients. For BC survivors, data is retrospective and reflects their financial situation at the end of the initial treatment. Details of the recruitment process and its results (including response rate) are available elsewhere [14] (preprint).

### Variables and measurements

#### Main outcome: financial toxicity (FT)

In principle, the cost of care can lead to objective financial burden that in turn can lead to subjective financial distress and FT is the potential consequence of this process [3]. While instruments to measure FT have been developed, their use is currently very limited. None of the three specific instruments for FT in cancer patients has been used or validated in LMICs [3, 15]. In this paper, we used parameters from a previous study of FT in Nepal [15]—another LMIC—and adapted these using experience acquired from a previous study of BC patients in Vietnam [2] to examine the issue of FT in Vietnam. As such, we defined those who could not afford the costs of care with their liquid assets (i.e. cash, savings and shares that could be readily converted to cash) and were obliged to resort to sale of illiquid assets, borrowing money from others or terminating treatment as those who experienced ‘financial toxicity’. This definition reflects the two main domains of FT which are (i) the use of active and passive financial resources (e.g. use of illiquid assets) and (ii) the coping behaviours that patients adopt to deal with the cost of care (e.g. borrowing money or terminating treatment) [3]. Questions used to measure FT are presented in Supplementary file 1 (ESM 1).

We assume that higher objective financial burden led to higher financial subjective distress and as such increase the degree of FT. Hence, we measured objective financial burden by the amount of money that exceeded patients’ ability to pay—the ‘deficit’—and use this to assess the degree of FT. A zero deficit means no FT and the higher the deficit, the more severe the FT. As the time that survivors finished their initial treatment ranged from 2005 to 2019, all amounts were adjusted from the original price year to the 2019 price year using a gross domestic product deflator index for Vietnam [16].

#### Covariates

As FT is the direct result of the imbalance between ability to pay and cost of treatment, the sociodemographic and clinical-related variables that are normally associated with either of these two factors, based on previous studies in Vietnam or elsewhere [4, 7, 11, 12], were included as covariates. The sociodemographic characteristics considered were ‘occupation’, ‘marital status’, ‘household monthly income’ and ‘coverage rate of HI’. The clinical characteristics considered were ‘stage of cancer at diagnosis’, ‘duration of treatment’ (in months), ‘treatment status’ (patients vs survivors), ‘year of treatment’ and ‘relapse status’.

### Data analysis

Descriptive statistics (mean and standard deviation for continuous variables, percentages for discrete variables) were used to describe the sociodemographic and clinical-related characteristics of the respondents. Descriptive statistics were also used to describe the deficit and the proportion of respondents who experienced different forms of FT.

The deficit (used to assess the degree of FT) contains many observations where the value was equal to zero. To deal with this distribution, we used the Cragg hurdle model [17] instead of the common ordinary least square regression model to assess the determinants of FT level. This is a more appropriate econometric approach to deal with this type of data [18, 19]. The Cragg hurdle model involves a two-step estimation procedure: (i) a Probit regression model examines the likelihood of experiencing FT; (ii) conditional of FT being experienced, a truncated normal regression model examines the extent of FT experienced. This model is designed to allow for the possibility that different factors might affect each stage of the estimation or the same factors might affect in a different way [18, 19]. In other words, factors affect patient’s probability of experiencing FT and factors that push the patients further into FT might be different or if the same, might have different levels of effect.

## Results

The combined online and hospital-based survey yielded 309 observations of which 107 (34.6%) were BC patients and 202 (65.4%) were BC survivors. The sociodemographic and clinical-related characteristics of respondents are presented in Table 1. The mean age (standard deviation (SD)) of patients/survivors was 48.1 (10.4) years. The majority of the sample was married (77%), working (69.5%, either full-time or self-employed) well educated (77.2% completed at least high school education) and possessed a HI with an 80% coverage rate (75.7%). At diagnosis, around two-thirds of the respondents had been diagnosed with stage II breast cancer while late-stage diagnosis (stages III and IV) accounted for 13.9%. The median duration of treatment (from the point of getting diagnosis to being discharged from the hospital) was 8 months.

**Table 1:** Sociodemographic and clinical-related characteristics of respondents

Characteristics (*n*=309)	Number of respondents	Percentage
Age (in years): mean (SD)	48.1 (10.4)	-
Level of education		
Completed at least secondary education	70	22.8
Completed high school education	57	18.6
Completed undergraduate degree	160	52.1
Completed graduate degree	20	6.5
Marital status		
Single/separated/divorce/widow	70	23.0
Married	235	77.0
Occupation		
Unemployed/student/homemaker	42	13.8
Full-time employee	133	43.6
Self-employed	79	25.9
Retired	51	16.7
Household monthly income (in Vietnamese Dong (VND))		
≤ 3,000,000 VND (~$129)	43	14.5
3,000,001–6,000,000 VND (~$130–259)	57	19.2
6,000,001–9,000,000 VND (~$260–389)	31	10.4
9,000,001–12,000,000 VND (~$390–518)	75	25.3
>12,000,000 VND (~$518)	91	30.6
Coverage rate of health insurance		
80%	227	75.7
95%	31	10.3
100%	42	14.0
Stage of cancer at diagnosis		
Stage 0/I	68	22.0
Stage II	185	59.9
Stage III	38	12.3
Stage IV	5	1.6
Do not know/Do not remember	13	4.2
Duration of treatment (in months): median (IQR)	8.0 (6.0–11.0)	-

*SD*, standard deviation; *IQR*, interquartile range; *VND*, Vietnamese Dong; *$*, United States Dollar

Currency exchange rate in October 2020: $1 = 23,176 VND

**Fig. 1:**
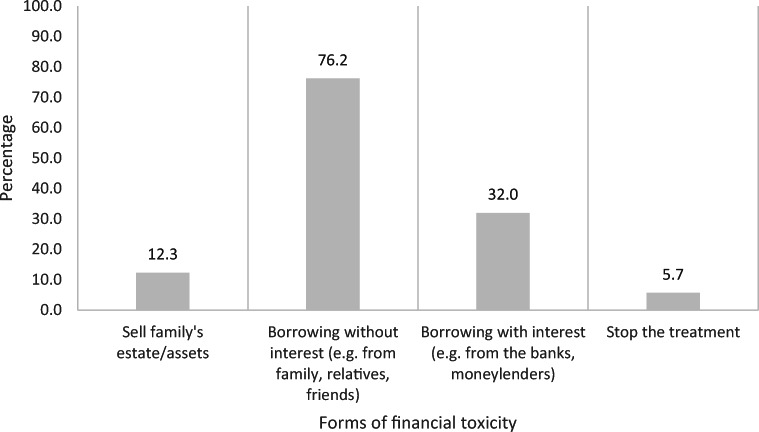
Forms of financial toxicity experienced by breast cancer patients/survivors due to treatment cost

Nearly half of the respondents (41%) reported that they could not afford the treatment with their liquid assets (e.g. cash, shares, savings) and experienced FT as a result. Respondents could make recourse to more than one source of support. Most often they made recourse to family members, relatives, and/or friends to borrow money without interest (76.2%), followed by borrowing money with interest from the banks and/or moneylenders (32.0%) and the sale of family land/estate/assets (12.3%) (Fig. 1). Besides, 5.7% of respondents reported that they decided to stop the treatment when the costs of care went above their ability to pay.

**Fig. 2:**
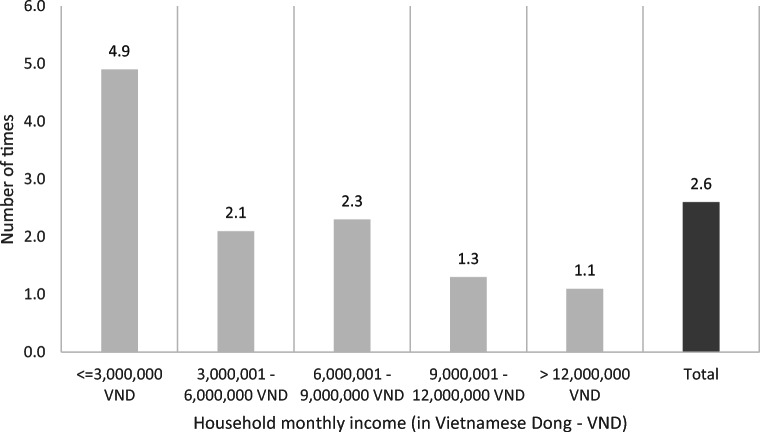
The mean number of times the deficit is greater than respondents’ annual household income

The mean deficit (amount of money that exceeds respondents’ ability to pay) was 153 million Vietnamese Dong (VND) (~$6602) with a wide range from 2.42 million VND to 1358 million VND (~$104–58,413). On average, the deficit was 2.6 times greater than patients’ household annual income (ranged from 0.06 to 25 times). The ratio of deficit to income was greatest among the group having least income. Indeed, respondents with household monthly income less than 3 million VND (~$129) compared with those with more than 12 million VND (~$518) faced a deficit to income ratio of approximately 5 and 1.1, respectively (Fig. 2). The difference was statistically significant (Kruskal Wallis test, *χ*^2^=14.563, *p*=0.0057).

Table 2 presents the results (as marginal effects) of the hurdle model. Each additional month of being in the treatment was associated with an extra 1.75 million VND (~$76) in the patients’ deficit (95% CI: 1.21–2.28, *p*<0.001). Diagnosed at stage II or III of BC was associated with 16.0 and 18.0 million VND (~$690–777) more in the deficit compared with those was diagnosed at stage 0/I, respectively (95% CI: 3.98–28.1, *p*=0.009 and −0.03–36.1, *p*=0.05 respectively). Being retired, being married, being in the group of survivors and having a HI with a 100% coverage rate were associated, respectively, with 21.0, 16.8, 18.1 and 12.2 million VND ($906, $725, $781 and $526) less in the deficit compared with the corresponding reference groups (all *p*<0.05, Table 2).

**Table 2:** Hurdle model analyses of factors influence the degree of financial toxicity (deficit)

	Marginal effect^a^	95% CI
Duration of treatment (in months)	1.75	1.21–2.28**
Stage of cancer at diagnosis		
Stage 0/I^ref^	-	-
Stage II	16.0	3.98–28.1*
Stage III	18.0	−0.03–36.1*
Stage IV	8.0	−15.0–31.0
Do not know/do not remember	−12.6	−24.8–−0.50*
Occupation		
Unemployed/student/homemaker^ref^	-	-
Full-time employee	−0.8	−21.4–19.8
Self-employed	−12.9	−32.4–6.6
Retired	–21.0	–41.9**–−**0.2*
Marital status		
Single/separated/divorce/widow^ref^	-	-
Married	**−**16.8	–31.1**–−**2.5*
Household monthly income (in Vietnamese Dong (VND))		
≤ 3,000,000 VND (~$129)^ref^	-	-
3,000,001–6,000,000 VND (~$130–259)	12.2	−11.8–36.2
6,000,001–9,000,000 VND (~$260–389)	2.6	−23.9–29.0
9,000,001–12,000,000 VND (~$390–518)	−8.2	−29.9–13.4
>12,000,000 VND (~$518)	–21.2	–42.6–0.2
Coverage rate of health insurance		
80%^ref^	-	-
95%	1.1	−9.0–11.2
100%	**−**12.2	**−**21.2**–−**3.2*
Year of treatment	−1.2	−4.3–1.9
Relapse status		
No^ref^	-	-
Yes	13.9	−8.1–35.9
Treatment status		
Patients undergoing treatment^ref^	-	-
Survivor	**−**18.1	**−**29.2–**−**6.9*

*ref*, reference group; *VND*, Vietnamese Dong; *$*, United States Dollar

Exchange rate in October 2020: $1 = 23,176 VND

^a^Marginal effect for factor levels is the discrete change from the base level (or reference group)

* *p*<0.05, **p<0.001

For completeness, results from the first part of the hurdle model which examines the likelihood of experiencing FT are reported in Supplementary file 2 (ESM 2). These show that being in stage III/IV of BC at diagnosis increase the chance FT occurred. In contrast, being retired, being in the group of survivors and having household monthly income higher than 12 million VND (~$518) lowered the chance of FT occurrence.

## Discussion

The analysis of data from this first study to investigate FT in Vietnam indicated that 41% of BC patients experienced FT as a result of treatment costs even though all respondents in the study possessed HI. It is currently not possible to compare our results with other studies as similar data in Vietnam has not been published. A previous study in three tertiary hospitals found that 71.8% of households with BC patients faced catastrophic health expenditure [11] though it is unclear whether they experienced FT due to catastrophic expenses. Studies from the USA, where most studies on FT have been reported, used data from the 2011–2016 Medical Expenditure Panel Survey and reported that 25.3% of cancer survivors faced material hardship (i.e. they needed to borrow money, incurred debt, declared bankruptcy or were unable to cover cost share) [20]. Other studies reported from 12 to 34% of cancer patients experienced FT [21–24]. In the only study, we found from LMICs, 100% of Nepalese patients with acute leukaemia faced FT in which they had to either ask for charity from the public, borrow with interest or sell property to fund treatment [15]. The fact that the sample comprised largely poor people, treatment costs for this type of cancer were high, and there was no national HI system may have contributed to this extremely high rate [15]. Nevertheless, the FT that (breast) cancer patients in Vietnam face is profound and there is a need to give priority to further research on this issue.

In order to pay for the treatment cost, BC patients/survivors depended firstly on their liquid assets (e.g. cash, shares, savings). When the liquid assets were exhausted, BC patients/survivors started experiencing FT in which they needed to borrow money from their family members, relatives and friends (76.2%), the banks and moneylenders (32%), and/or sell their family assets/estate (12.3%). The situation was similar with cancer patients in ASEAN where 67% asked for financial assistance from family/friends, 28% took personal loans and 20% had sold their assets [5]. In addition, 5.7% of respondents in our study said they stopped treatment due to their inability to afford it. This is not surprising, as noted by one BC patient/survivor in a previous qualitative study ‘If you have a house then you can sell your house, but if you don’t, then what can you sell to get money for treatments? Many people got hopeless and did not go to the hospital to be treated’ [2]. We did not have the opportunity to assess further the strategies used by respondents to deal with costs nor the psychological effects of funding care but literature from the USA and ASEAN suggests that patients/survivors may have a lower quality of life, opt to medication nonadherence, change lifestyle to save more money and delay or forego treatment [25–29].

The degree of FT was assessed through ‘deficit’—the objective financial burden arose when treatment costs exceed patients’ ability to pay. The mean deficit was 153 million VND (~ $6602). To put this in context, it was 2.6 times greater than the respondents’ household annual income and 4 times greater than the income per capita of Vietnam [30]. The deficit varied remarkably from 2.42 million VND to 1358 million VND (~$104–58,413) which was 0.06 and 5 times greater than the respondent’s annual household income, respectively. This divergence was influenced by several factors.

Firstly, the experience of FT was significantly associated with a later stage of BC at diagnosis, unemployed or self-employed and lower household monthly income. The last two factors were similar to the results from US studies [22, 23]. It is consistent with intuition that stage of cancer may influence the ability to pay as the later stage is associated with higher cost of treatment [7, 12] though this will be no linear as those at the late stage may be poor candidates for some types of aggressive treatment.

When FT occurred, the factors associated with its increased severity were longer duration of treatment and later stage of cancer at diagnosis though the effect of the latter was much higher than the former. Protective factors that helped reduce the deficit were being retired, married, in the group of survivors, and having a HI with a 100% coverage rate (0% coinsurance). Antiquity of treatment and relapse status were not significant, though this may in part be due to collinearity with survivor status. Possible reasons for the association between retirement and the deficit are the savings that retired person may have accumulated during their working life, the pension they received every month (working person may need to stop working while being treated; thus, loss of salary) and the reduction of coinsurance for health care services from 20 to 5%. In our study, the group of survivors had significantly higher education, household monthly income; lived in urban areas; were diagnosed at an earlier stage of cancer; and had shorter duration of treatment compared to the group of patients undergoing treatment [14]. This difference might in part explain why being in the survivor group was associated with lower degree of FT.

It is interesting to see the two factors ‘marital status’ and ‘household monthly income’ affect the risk of getting into FT and degree of FT differently. Having the household monthly income higher than 12 million VND (~$518) decreased the likelihood of experiencing FT but it was not associated with the degree of FT. Those with higher income perhaps can accumulate more liquid assets (cash, saving, stock etc.); thus, they might delay or even avoid the occurrence of FT. However, once FT occurred, monthly income may not play a significant role in controlling the FT perhaps because a relatively small amount of costs can actually be paid for from current income. In contrast, being married was not associated with FT occurrence but it helped lower the degree of FT. The long-term support in direct finance, in terms of sharing other household duties, and mental health of the patient’s partner might explain why being married helps protect the patients from falling deeper into FT once it occurred.

Financial support for cancer patients is limited in Vietnam as in many other LMICs where the priority lies on improving access to more routine/primary care services. The country’s HI system protects people to a certain extent, but the personal liability for care costs remains high at 20% or even 100% in case of services not covered by HI. Government benefits such as reduction of coinsurance from 20% to 0–5% or monthly allowance of 270,000–540,000 VND (~$12–24) are only provided for those who fall below the poverty line or elderly/unmarried persons in poor/near-poor households [31, 32]. Charity grants are rare as the country has only one dedicated foundation for this purpose: Supportive Fund for the Cancer Patients—Bright Future. Instead, patients/survivors are obliged to help each other through their self-established patient/survivor support clubs/groups following the Vietnamese proverb and moral tradition ‘The good leaves protect the worn-out leaves’. The lack of financial support leaves patients/survivors more vulnerable to adverse outcomes as mentioned above. Government and/or non-government organizations’ interventions such as offering loans at low or even 0% interest rates to cancer patients, facilitating return to work after treatment, and promotion of screening so that women can be diagnosed at an earlier stage of cancer may offer ways in which the financial impact of cancer can be mitigated.

This is the first study in Vietnam to report the detrimental effects of the excess OOP expenses for BC treatment on the patients and their families. It is also among the very few that comes from LMICs. The study provides novel and valuable insights about the forms and degree of FT faced by BC patients/survivors as well as the factors associated with the occurrence of FT and its severity. It offers an insight into the problem of ‘financial toxicity’ of cancer care in LMICs or at least for countries with similar health systems as Vietnam—with public-funded health care services but high co-payment from both the patients. (Breast) Cancer patients in LMICs, especially where cancer is diagnosed at a late stage, where there is no national screening programme, high direct and indirect costs of care and low coverage of HI, are likely to be more vulnerable to FT. Research on this topic and its associated factors in LMICs is urgently needed in order to facilitate policies that mitigate the effect of FT.

Apart from these strengths, the study also has some limitations. Firstly, the deficit was self-reported by the respondents and it was unable for us to cross-check that information with any other sources. As we interviewed the survivors regardless of their time of diagnosis, some respondents have finished their treatment for years; thus, recall bias was a possibility when we asked for the costs of diagnosis and initial treatment and financial well-being at the point of finishing these treatments. Future research which can afford prospective data collection can opt to recruit individuals at different stages of their cancer journey and follow them up to avoid recall bias. The definition of FT used in this study could only cover two out of the three domains related to subjective financial distress. The uncovered domain was the patients’ psychological response. We suggest other authors use the framework in the systematic review of Witte et al. [3] to unify the definition of FT (this article was not published when we designed our study). The self-designed questionnaire we used may hinder the comparison of this study with others although 70% of research on this topic also used self-designed questionnaires [3]. As the first study in Vietnam and for explanatory purposes, the study still provides valuable insight. However, moving forward, we acknowledge the need for a unified instrument and urge research to validate such an instrument to be conducted in countries around the world.

## Conclusions

Nearly half of BC patients/survivors experienced FT due to the cost of BC treatment. The deficit that occurred when OOP exceeded their ability to pay with liquid assets was on average 2.6–4 times greater than the respondents’ household annual income or income per capita of Vietnam. Some patients may be forced to stop the treatment due to its prohibitive cost. Unsurprisingly, a longer duration of treatment and a later stage at diagnosis is likely to push BC patients/survivors deeper into FT. The importance of early detection through screening for BC and easy access to treatment is self-evident. The FT that Vietnamese BC patients/survivors face due to BC treatment is profound, especially while financial support from government and/or non-government/charity organizations is scarce. Policies that mitigate this financial toxicity such as offering loans at low or zero interest rates, facilitating return-to-work programmes after treatment and promoting screening uptake for BC which offers the added benefit of improving outcomes are urgently needed.

## Supplementary information

ESM 1 (PDF 248 kb)

ESM 2 (PDF 247 kb)

## Data Availability

All data generated or analysed during this study are included in this published article. No additional data is available.

## Declarations

### Disclaimer

The funder of the study had no role in study design, data collection, data analysis, data interpretation or writing of the report. The corresponding author had full access to all the data in the study and had final responsibility for the decision to submit for publication.
