# Supplementary material for: Financial toxicity due to breast cancer treatment in low- and middle-income countries: evidence from Vietnam
Source: Support Care Cancer. 2021 Apr 16;29(11):6325–33. doi: 10.1007/s00520-021-06210-z (PMC8464564; doi:10.1007/s00520-021-06210-z)
Supplement: Supplementary file 1 — (PDF 248 kb) [file 520_2021_6210_MOESM1_ESM.pdf]

Supplementary material

Journal: Supportive Care in Cancer

Financial toxicity due to breast cancer treatment in low- and middle-income countries: evidence from Vietnam

Tran Thu Ngan[1 2 *], Hoang Van Minh[2], Michael Donnelly[1], Ciaran O'Neill[1]

[1] *Centre for Public Health, Queen's University Belfast, Belfast, United Kingdom*

[2] *Centre for Population Health Sciences, Hanoi University of Public Health, Hanoi, Vietnam*

* Correspondence to:

Tran Thu Ngan, MIH, PhD candidate

Postal address: Centre for Public Health, Queen's University Belfast, Belfast, United Kingdom

Email: ntran02@qub.ac.uk

Questions were used to determine the occurrence and degree of financial toxicity

Variable	Definition	Question
**Financial toxicity**	BC patients who could not afford the costs of care with their liquid assets (i.e., cash, savings and shares that could be readily converted to cash) and were obliged to resort to sale of illiquid assets, borrowing or terminating treatment were defined as experiencing ‘financial toxicity’.	B9, B11
**Deficit (degree of financial toxicity)**	The amount of money that exceed respondents’ ability to pay with their liquid assets	B10

Related questions extracted from the questionnaire[1]

**B6. What types of following health services have you received or receiving?**<br>*Ask for each row. If respondents answer “No” for B6, skip B7 and B8 of that row and move to the next row.*			**B7. If yes, what were the costs you paid for that service?**	**B8. Did the health insurance pay for any part of that service’s cost?**	
	**Yes**	**No**		**Yes**	**No**
Health services relate to examination and diagnosis (e.g. *clinical breast examination, ultrasound, mammography, lab test (e.g.: blood test, etc.), biopsy etc.)*					
Lumpectomy *(breast-conserving surgery, only the part of the breast containing the cancer is removed)*					
Mastectomy *(complete removal of all breast gland tissue)*					
Breast reconstruction surgery *(using an implant or your own tissue)*					
Chemotherapy					
Radiotherapy					
Targeted therapy					
Other (please specify)					

B9. Did your family have enough money to pay for all those expenses mentioned above (in B7)?

1. Yes, enough money → go to B12
2. No, not enough money

[1] This version of the questionnaire was the one used in hospital-based survey with instruction for interviewers (online version has the same content but different instruction to facilitate the self-administrative responses from respondents). The questionnaire was first designed in English. Data collection used questionnaire in Vietnamese.

B10. How much was the missing? ________________________________

(Write 99 if don't remember)

B11. What was the solution for the missing money? Multiple choice

1. Sell family's land/estate/asset
2. Borrowing without interest (e.g., from relatives, friends…)
3. Borrowing with interest (e.g., from the bank, moneylenders…)
4. Withdraw from savings/stock sale
5. Stop the treatment

97. Other (specify): ____

99. Don't know/Don't remember

98. Refuse to answer
