# Supplementary material for: Financial toxicity due to breast cancer treatment in low- and middle-income countries: evidence from Vietnam
Source: Support Care Cancer. 2021 Apr 16;29(11):6325–33. doi: 10.1007/s00520-021-06210-z (PMC8464564; doi:10.1007/s00520-021-06210-z)
Supplement: Supplementary file 2 — (PDF 247 kb) [file 520_2021_6210_MOESM2_ESM.pdf]

Supplementary material

Journal: Supportive Care in Cancer

Financial toxicity due to breast cancer treatment in low- and middle-income countries: evidence from Vietnam

Tran Thu Ngan[1 2 *], Hoang Van Minh[2], Michael Donnelly[1], Ciaran O'Neill[1]

[1] *Centre for Public Health, Queen's University Belfast, Belfast, United Kingdom*

[2] *Centre for Population Health Sciences, Hanoi University of Public Health, Hanoi, Vietnam*

* Correspondence to:

Tran Thu Ngan, MIH, PhD candidate

Postal address: Centre for Public Health, Queen's University Belfast, Belfast, United Kingdom

Email: ntran02@qub.ac.uk

Results from the first part of the Cragg double hurdle model (assess factors influence the occurrence of financial toxicity)

	Marginal effect[a]	95% CI
Stage of cancer at diagnosis		
Stage 0/I[ref]	-	-
Stage II	-1.1	-0.23 – 0.21
Stage III	**-0.20**	**-0.39 – -0.005***
Stage IV	**-0.35**	**-0.64 – -0.06***
Do not know/Do not remember	0.03	-0.24 – 0.30
Occupation		
Unemployed/Student/Homemaker[ref]	-	-
Full-time employee	0.14	-0.05 – 0.34
Self-employed	0.11	-0.09 – 0.30
Retired	**0.29**	**0.08 – 0.50***
Marital status		
Single/separated/divorce/widow[ref]	-	-
Married	0.11	-0.03 – 0.25
Household monthly income (in Vietnamese Dong - VND)		
≤ 3,000,000 VND (~$129)[ref]	-	-
3,000,001 – 6,000,000 VND (~$130-259)	-0.03	-0.25 – 0.19
6,000,001 – 9,000,000 VND (~$260-389)	-0.07	-0.34 – 0.20
9,000,001 – 12,000,000 VND (~$390-518)	0.09	-0.13 – 0.32
>12,000,000 VND (~$518)	**0.33**	**0.11 – 0.55***
Year of treatment	0.004	-0.02 – 0.03
Relapse status		
No[ref]	-	-
Yes	-0.17	-0.43 – 0.09
Treatment status		
Patients undergoing treatment[ref]	-	-
Survivors	**0.22**	**0.09 – 0.34****

ref: Reference group | VND: Vietnamese Dong | $: United States Dollar
Exchange rate in October 2020: $1 = 23,176 VND
[a] Marginal effect reflect changes in probability of NOT experiencing financial toxicity from the base level (or reference group)
** p<0.05, ** p<0.001*
